# Dyslipidemia and the Prevalence of Hypertension: A Cross-Sectional Study Based on Chinese Adults Without Type 2 Diabetes Mellitus

**DOI:** 10.3389/fcvm.2022.938363

**Published:** 2022-07-07

**Authors:** Wenke Cheng, Jingqi Zhuang, Siwei Chen

**Affiliations:** ^1^Medical Faculty, University of Leipzig, Leipzig, Germany; ^2^Department of Admission and Follow-Up, Lintong Rehabilitation and Recuperation Center, Xian, China; ^3^Department of Cardiovascular Medicine, The Third Hospital of Nanchang, Nanchang, China

**Keywords:** dyslipidemia, lipid, atherosclerotic indices, hypertension, cross-sectional study

## Abstract

**Background:**

In clinical practice, it is frequently observed that patients with hypertension often coexist with dyslipidemia. However, studies on atherosclerotic indices and the prevalence of hypertension are still limited. The purpose of this study was to assess the relationship between atherosclerotic indices and the prevalence of hypertension in Chinese adults without type 2 diabetes mellitus.

**Methods:**

In this paper, a cross-sectional study was conducted based on 117,056 adults in 11 Chinese cities (Shanghai, Beijing, Wuhan, Suzhou, Shenzhen, Changzhou, Nantong, Guangzhou, Hefei, Nanjing, and Chengdu) from 2010 to 2016. Besides, the raw data was obtained from the public database (www.Datadryad.org), while eight atherosclerosis indices namely the atherogenic coefficient (AC), Castelli's risk index I (CRI-I) and II (CRI-II), the atherogenic index of plasma (AIP), the cholesterol index, the lipoprotein combined index (LCI), non-high-density lipoprotein cholesterol (non-HDL-C) and triglycerides/high-density lipoprotein cholesterol (TG/HDL-C) were analyzed in this study. Apart from that, two groups of continuous variables were measured using the Mann-Whitney test, and categorical variables were analyzed using the Chi-square test. Differences between multiple groups of continuous variables were investigated using Kruskal-Wallis one-way analysis of variance (ANOVA) and Dunn's test. Furthermore, Spearman correlation analysis and multivariate logistic regression analyses were performed to assess the relationship between atherosclerotic indices and blood pressure levels, and the prevalence of hypertension, respectively. The results of multivariate logistic regression analyses were expressed as the odds ratio (OR) and their corresponding 95% confidence intervals (CIs). Moreover, the receiver operating characteristic (ROC) curve was depicted to further analyze the predictive value of the atherosclerotic indices on the prevalence of hypertension.

**Results:**

The atherosclerosis indices were higher in the hypertensive population compared to those in the normotensive population. Meanwhile, systolic blood pressure (SBP) and diastolic blood pressure (DBP) were linearly and positively correlated with atherosclerotic indices. In addition, multivariate logistic regression analysis showed that the cholesterol index and non-HDL-C were observed to be positively associated with the prevalence of hypertension (*p for trend* < 0.05). Moreover, the prevalence of hypertension increased by 3.7% (OR: 1.037; 95% CI: 1.009-1.065; *p* = 0.009) and 6.1% (OR: 1.06; 95% CI: 1.033–1.091; *p* < 0.001), respectively, as per 1-standard deviation (SD) increase in the cholesterol index and non-HDL-C. Beyond that, ROC analysis demonstrated that the cholesterol index and non-HDL-C have a good predictive value for the prevalence of hypertension in women, with under the ROC curve (AUC) of 0.659 and 0.684 and cut-off values of 47.94 and 134.34 mg/dl, accordingly.

**Conclusions:**

In Chinese adults without type 2 diabetes mellitus, atherosclerotic indices were significantly higher in hypertensive populations compared with those in normotensive populations, regardless of hypertension levels. Meanwhile, SBP and DBP were linearly and positively related to atherosclerotic indices. Besides, the cholesterol index and non-HDL-C were independent risk factors for the prevalence of hypertension, and they could be adopted for effectively predicting the prevalence of hypertension in women.

## Introduction

Hypertension is one of the most common risk factors for cardiovascular disease (CVD). Besides, there are more than one billion people worldwide suffering from it, and the number is still increasing. Thus, hypertension becomes a serious public health problem globally (1). Currently, approximately 23.2% of adults in China suffer from hypertension and only 15.3% of cases are under control, which directly contributes to the fact that hypertension-related cardiovascular disease remains the leading cause of death among Chinese adults (2, 3). Therefore, early primary prevention and identification of risk factors for hypertension are essential to reduce the public health burden.

Typically, dyslipidemia is defined as elevated plasma lipids (low-density lipoprotein cholesterol, LDL-C; total cholesterol, TC; triglycerides, TG) or reduced levels of high-density lipoprotein cholesterol (HDL-C) (4). It adversely affects the functional and structural properties of arteries and promotes atherosclerosis (5), which is a common risk factor for most cardiovascular diseases such as coronary artery disease, strokes, and myocardial infarction (6, 7). However, reliance on LDL-C or HDL-C alone is inefficient for the diagnosis of cardiovascular disease risk stratification (8). Therefore, the combination of multiple lipid parameters might predict CVD risk more accurately than simple lipid parameters. Over the past 20 years, several unconventional lipid ratios: atherosclerosis indices, including the atherogenic coefficient (AC), Castelli's risk index I (CRI-I) and II (CRI-II), the atherogenic index of plasma (AIP), the cholesterol index, the lipoprotein combined index (LCI), non-HDL-C and TG/HDL-C, have been proposed. Meanwhile, many studies have confirmed that atherosclerotic indices is conducive to the predication of CVD risk and atherosclerosis (8–11).

In clinical practice, it is frequently observed that patients with hypertension often coexist with dyslipidemia (12). Therefore, here, it was speculated that the atherosclerotic indices may be equally predictive of the prevalence of hypertension. Currently, studies on atherosclerotic indices and the prevalence of hypertension are still limited (13, 14). Furthermore, to better serve the clinic, whether there are differences in the predictive value of hypertension prevalence between different atherosclerotic indices should be further evaluated. To address these issues, the purpose of this study was to assess the relationship between atherosclerotic indices and the prevalence of hypertension in Chinese adults without type 2 diabetes mellitus.

## Methods

### Study Design and Data Extraction

Here, a cross-sectional study was conducted with a secondary analysis of participants from 32 health screening centers in China from 2010 to 2016. Participant-related medical records were obtained from a computerized database (www.Datadryad.org) established by Rich Healthcare Group. The original data were provided by Chen et al. (15). A total of 211,833 Chinese adults without type 2 diabetes mellitus from 11 Chinese cities (Shanghai, Beijing, Wuhan, Suzhou, Shenzhen, Changzhou, Nantong, Guangzhou, Hefei, Nanjing, and Chengdu) were recruited for the original study that aimed to investigate the association of body mass index and age with the prevalence of diabetes. Specifically, all participants filled out a detailed questionnaire, including demographics, lifestyle and family history of chronic disease, on their first visit to the health screening center. Besides, biochemical parameters, such as TG, TC, LDL-C, HDL-C, fasting plasma glucose (FPG), blood urea nitrogen (BUN), serum creatinine (Scr), aspartate, aminotransferase (AST), and alanine aminotransferase (ALT) were measured using a uniform automated analyzer (Beckman 5800). Other than that, the body mass index (BMI) was calculated by dividing body weight (kg) by the square of height (m^2^). All data were collected uniformly during a standardized process.

As Chen et al. have waived all copyrights and ownership associated with the raw data, we were able to reuse these data for analysis without infringing on the authors' rights (15). In addition, the original study was approved by the Rich Healthcare Group Review Board; the information was retrieved retrospectively, and the data was anonymized without infringing on the rights of the participants. This study is observational and in compliance with *the Declaration of Helsinki*, while the Rich Healthcare Group Review Board waived the requirement for informed consent (16).

### Study Population

**Figure 1:**
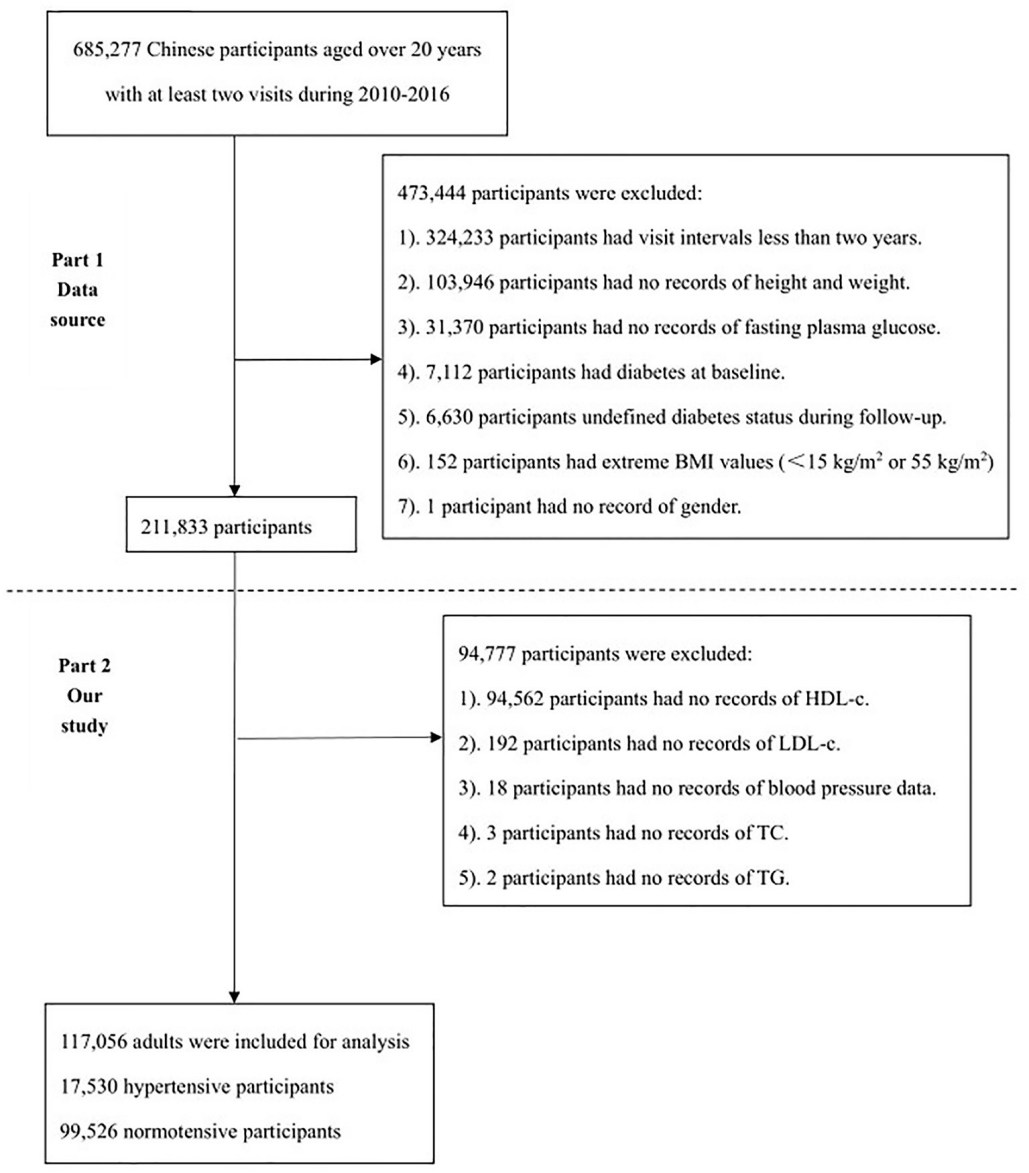
The flowchart of study. The first part is the flowchart of the original study and the second part is the flowchart of the current study.

Here, a secondary analysis on the results given by Chen et al. is made. The dataset was downloaded from a public database (www.Datadryad.org) and contained baseline information of 211,833 participants. Therefore, the inclusion criteria were consistent with the original study, with non-type 2 diabetic adults aged between 20 and 99 years and having at least two visits between 2010 and 2016, along with complete information on participants' height, weight and FPG. As shown in Figure 1, the selection of participants consisted of two parts. The first part is the flowchart of the original study with a total of 211,833 participants included, and the specific reasons for exclusion are listed below: 1). 324,233 participants had visit intervals <2 years; 2). 103,946 participants had no records of height and weight; 3). 31,370 participants had no records of fasting plasma glucose; 4). 7,112 participants had diabetes at baseline; 5). 6,630 participants were not defined as diabetes status during follow-up; 6). 152 participants had extreme BMI values (<15 kg/m^2^ or 55 kg/m^2^); 7). 1 participant had no record of gender. The second part is the flowchart of the current study, where only participants with complete information on lipid profile were included. For this reason, 94,777 participants were excluded for the following specific reasons: 1). 94,562 participants had no records of HDL-C; 2). 192 participants had no records of LDL-C; 3). 18 participants had no records of blood pressure data; 4). 3 participants had no records of TC; 5). 2 participants had no records of TG. Finally, 117,056 adults were included, featuring 17,530 hypertensive participants and 99,526 normotensive participants.

### Exposure of Interest and Outcome Measures

The exposure of interest was atherosclerotic indices including AC, CRI-I, CRI-II, AIP, LCI, the cholesterol index, non-HDL-C, and TG/HDL-C that were calculated by the following formulas (8, 10, 17): AC = (TC-HDL-C)/HDL-C; AIP = Log(TG/HDL-C); non-HDL-C = TC-HDL-C; CRI-I = (TC/HDL-C); CRI-II = (LDL-C/HDL-C); LCI = TC^*^TG^*^LDL-C/HDL-C; cholesterol index = LDL-C-HDL-C (TG <400mg/dl) or LDL-C-HDL-C+TG/5 (TG≥ 400mg/dl).

The study outcome was the prevalence of hypertension. Blood pressure values were obtained by trained staff using a standard mercury sphygmomanometer in a quiet environment. Hypertension was defined as systolic blood pressure (SBP) ≥140 mmHg or diastolic blood pressure (DBP) ≥90 mmHg according to the *Chinese Guidelines for the Prevention and Treatment of Hypertension (2018 edition)* (18). Meanwhile, the hypertensive group was further classified into mild hypertension (class I; SBP 140-159 mmHg or DBP 90-99 mmHg), moderate hypertension (class II; SBP 160-179 mmHg or DPB 100-109 mmHg), and severe hypertension (class III; SBP ≥180 mmHg or DBP ≥110 mmHg). While normotension was defined as SBP <140 mmHg and DBP <90 mmHg.

### Statistical Analyses

All continuous variables were skewed distribution and therefore expressed as medians (interquartile range, IQR), while categorical variables were expressed as percentages. Besides, two groups of continuous variables were analyzed using the Mann-Whitney test, and categorical variables were measured by performing the Chi-square test. Apart from that, correlation analysis of SBP, DBP and atherosclerotic indices was performed by the Spearman test. In addition, the overall participants were further classified into normal, grade I, grade II, and grade III groups according to baseline blood pressure levels, and multiple comparisons of atherosclerotic indices were conducted by Kruskal-Wallis one-way ANOVA and Dunn's test, whereas univariate and multivariate logistic regression analyses were made to assess the relationship between atherosclerotic indices and the prevalence of hypertension. The results of both univariate and multivariate logistic regression analyses were expressed as the odds ratio (OR) and their corresponding 95% confidence intervals (CIs). Statistically significant atherosclerotic indices were then brought into receiver operating characteristic (ROC) curve analysis to further analyze their predictive value for the prevalence of hypertension. Moreover, the Youden's J index determined the cut-off values for the atherosclerotic indices and the prevalence of hypertension, and statistical analysis was carried out using SPSS 26.0 and GraphPad 9.0. A two-tailed *p-*value less than 0.05 indicates statistical difference.

## Results

Demographic data and baseline characteristics of the overall participants are shown in Table 1. A total of 117,056 participants with a median age of 41 (IQR, 34-53) years were recruited, when 54,099 (46.2%) women were involved. Based on baseline blood pressure levels, all participants were classified into normotensive and hypertensive groups. Compared to the normotensive population, the hypertensive group was older, and had a higher proportion of current smokers and drinkers, higher levels of the BMI, FPG, AST, ALT, BUN and Scr, while a lower proportion of women and family history of diabetes. In terms of the conventional lipid profile, the hypertensive group was featured with higher levels of TC, LDL and non-HDL-C and lower levels of HDL-C, while there were no significant differences in TG. In addition, in the hypertensive group, all reported atherosclerotic indices were higher than those in the normotensive group.

**Table 1:** Baseline information of the overall population^*^.

	**Total (*n* = 117,056)**	**Normotension (*n* = 99,526)**	**Hypertension (*n* = 17,530)**	***p*-value**
Age(years)	41 (34–53)	39 (33–50)	53 (41–63)	<0.001
Women (%)	54,099 (46.2)	48,304 (48.5)	5,795 (33.1)	<0.001
Current smoker (%)	6,674 (5.7)	5,520 (5.5)	1,154 (6.6)	<0.001
Current drinker (%)	872 (0.7)	651 (0.6)	221 (1.3)	<0.001
Family history of diabetes (%)	2,651 (2.3)	2,367 (2.4)	284 (1.6)	<0.001
BMI (kg/m^2^)	23.2 (21–25.5)	22.9 (20.8–25.1)	25.1 (23–27.3)	<0.001
FPG (mmol/L)	5.0 (4.61–5.35)	4.95 (4.6–5.3)	5.19 (4.8–5.62)	<0.001
ALT (U/L)	18.2 (13–27.8)	18 (12.9–26.8)	22 (15.7–33)	<0.001
AST (U/L)	22.0 (18.7–26.9)	21.8 (18.2–26)	24.2 (20.7–29.7)	<0.001
BUN (mmol/L)	4.57 (3.85–5.4)	4.53 (3.81–5.35)	4.78 (4.06–5.63)	<0.001
Scr (μmol/L)	71.3 (59.3–83.0)	70.5 (58.8–82.4)	75.8 (64.23–86.0)	<0.001
**Conventional Lipid Profiles**				
TC (mg/dl)	181.70 (159.67–204.90)	179.77 (158.51–202.97)	191.75 (168.56–216.50)	<0.001
TG (mg/dl)	97.46 (67.34–147.1)	97.46 (67.34–147.1)	97.46 (67.34–147.1)	0.719
LDL-C (mg/dl)	104.38 (88.53–121.78)	103.22 (87.76–120.62)	110.95 (93.94–128.74)	<0.001
HDL-C (mg/dl)	51.80 (45.23–59.92)	52.19 (45.23–59.92)	51.42 (44.46–59.15)	<0.001
**Atherogenic indices**				
AC	2.43 (1.95–3.1)	2.38 (1.91–3.04)	2.72 (2.21–3.46)	<0.001
AIP	0.28 (0.1–0.47)	0.28 (0.10–0.46)	0.29 (0.11–0.48)	<0.001
CRI-I	3.43 (2.95–4.10)	3.38 (2.91–4.04)	3.72 (3.21–4.46)	<0.001
CRI-II	1.98 (1.64–2.44)	1.95 (1.61–2.41)	2.16 (1.82–2.63)	<0.001
Cholesterol index (mg/dl)	51.80 (35.57–71.13)	50.26 (34.41–69.59)	59.92 (43.30–78.87)	<0.001
LCI	36,042 (22,238–59,550)	35,096 (21,684–58,094)	41,557 (25,854–67,465)	<0.001
TG/HDL-c	1.90 (1.26–2.92)	1.89 (1.26–2.91)	1.94 (1.30–2.98)	<0.001
Non-HDL-c (mg/dl)	127.96 (108.25–151.16)	126.03 (106.70–148.84)	138.40 (117.53–162.37)	<0.001

*Continuous data are expressed as median (interquartile range) due to the skewed distribution.

*FPG, fasting plasma glucose; TG, triglycerides; TC, total cholesterol; HDL-C, high-density lipoprotein cholesterol; LDL-C, low-density lipoprotein cholesterol; Scr, Serum creatinine; BUN, blood urea nitrogen; ALT, alanine aminotransferase; AST, aspartate aminotransferase; BMI, Body mass index; AC, atherogenic coefficient; AIP, atherogenic index of plasma; LCI, lipoprotein combine index; CRI, Castelli risk indices*.

### Correlation of SBP, DBP and Atherosclerotic Indices

Spearman correlation analysis revealed a positive correlation between the SBP and AC (Spearman correlation coefficient (rho) rho = 0.218, *p* < 0.001), AIP (rho = 0.037, *p* < 0.001), CRI-I (rho = 0.218, *p* < 0.001), CRI-II (rho = 0.196, *p* < 0.001), the cholesterol index (rho = 0.188, *p* < 0.001), LCI (rho = 0.124, *p* < 0.001), TG/HDL-C (rho = 0.031, *p* < 0.001), and non-HDL-C (rho = 0.213, *p* < 0.001).

Similarly, DBP was positively correlated with AC (rho = 0.229, *p* < 0.001), AIP (rho = 0.046, *p* < 0.001), CRI-I (rho = 0.229, *p* < 0.001), CRI-II (rho = 0.205, *p* < 0.001), the cholesterol index (rho = 0.192, *p* < 0.001), LCI (rho = 0.131, *p* < 0.001), TG/HDL-C (rho = 0.046, *p* < 0.001), and non-HDL-C (rho = 0.216, *p* < 0.001).

### Differences in Atherosclerotic Indices Between Groups With Different Blood Pressure Levels

**Figure 2:**
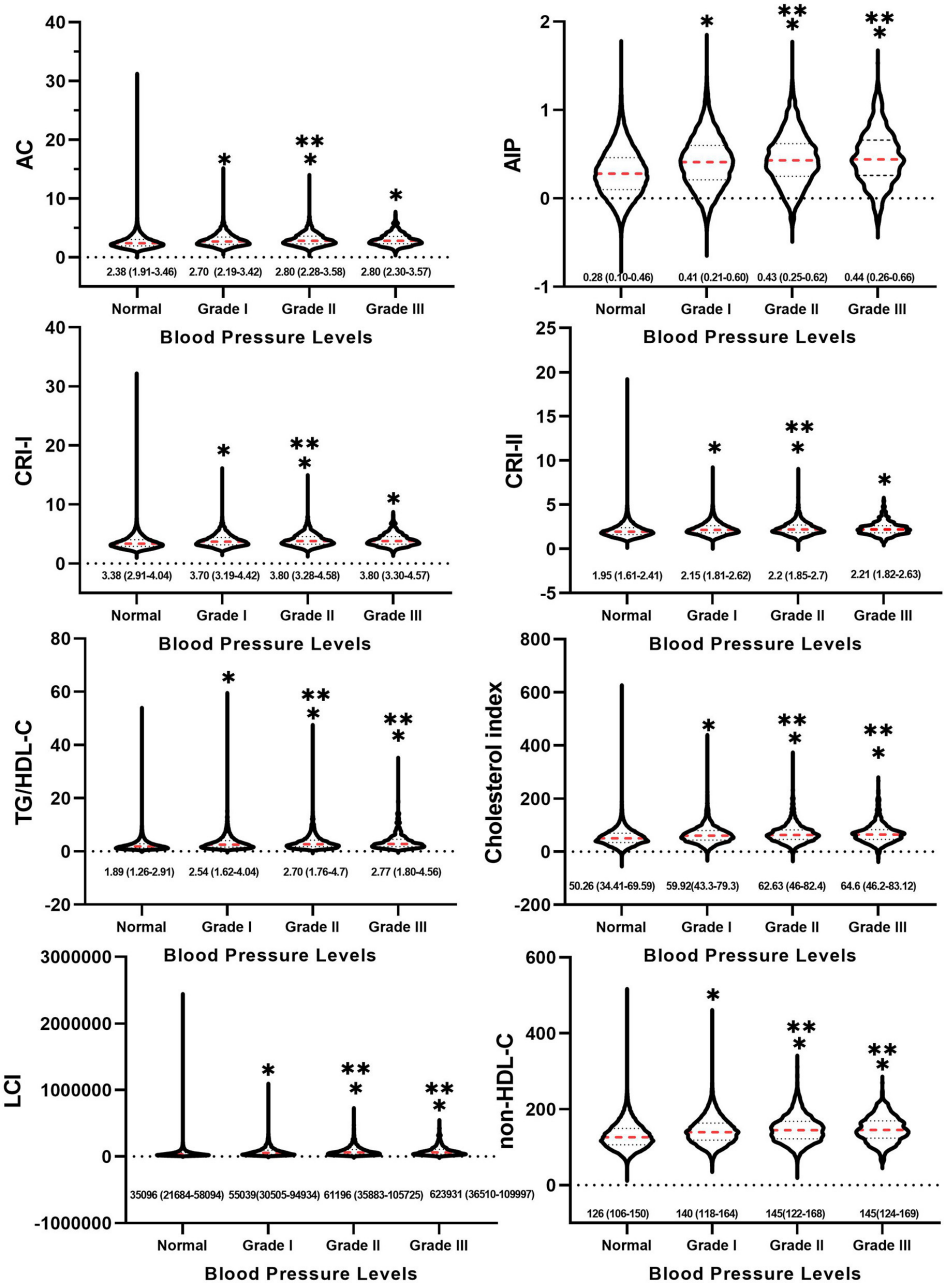
Between-group differences in atherosclerotic indices at different blood pressure levels. * indicates Grade I, Grade II and Grade III compared with normal group with *p* < 0.05. ** indicates Grade II and Grade III compared with Grade I with *p* < 0.05.

As displayed in Figure 2, the atherosclerotic indices were obviously higher in the hypertensive population compared to those in the normotensive group, regardless of high blood pressure levels (*p* < 0.05). In addition, among the hypertensive population, AC, CRI-I, and CRI-II were significantly higher in the grade 2 group than in the grade 1 group (*p* < 0.05), whereas there were no significant differences between the grade 3 and grade 1 groups. Meanwhile, AIP, TG/HDL-C, the cholesterol index, LCI, and non-HDL-C were noticeably higher in the grade 2 and grade 3 groups than in the grade 1 group (*p* < 0.05), while there was no significant difference between the grade 3 and grade 2 groups.

### Multivariate Logistic Regression Analysis of Atherosclerotic Indices and Hypertension Prevalence

In the crude model (univariate analysis), no variables were adjusted. In Model 1, age and sex were adjusted. In Model 2, the Model plus BMI was adjusted. In Model 3, the maximum variable was adjusted, that is, Model 2 plus FPG, ALT, AST, BUN, Scr, smoking status, drinking status, and family history of diabetes were adjusted.

The values of atherosclerosis indices were divided into quartiles, with the first quartile (Q1) being considered as the reference group, as shown in Table 2. In the crude model, all reported atherosclerotic indices were positively associated with the risk of hypertension (*p for trend* < 0.001); both expressed per quartile of atherosclerotic indices and per 1-standard deviation (SD) increase in atherosclerotic indices (*p* < 0.001). After adjusting for age and sex, AC, CRI-I, CRI-II, the cholesterol index, LCI, and non-HDL-C remained positively related to hypertension (*p for trend* < 0.001). Meanwhile, AC, CRI-I, CRI-II, the cholesterol index, LCI, and non-HDL-C remained positively associated with prevalence of hypertension after adjusting for age and sex (Model 2), as well as after additional adjustment for the BMI (Model 3). However, in Model 4, when the maximum variables were adjusted, only the cholesterol index and non-HDL-C were observed to be positively associated with the prevalence of hypertension (*p for trend* < 0.05). Moreover, the prevalence of hypertension increased by 3.7% (OR: 1.037; 95% CI: 1.009-1.065; *p* = 0.009) and 6.1% (OR: 1.06; 95% CI: 1.033-1.091; *p* < 0.001), respectively, as per SD increase in the cholesterol index and non-HDL-C.

**Table 2:** Multivariate logistic regression models for atherosclerotic indices and hypertension prevalence.

	**Crude model**		**Model 1**		**Model 2**		**Model 3**	
	**OR (95% CI)**	***p-*value**	**OR (95% CI)**	***p-*value**	**OR (95% CI)**	***p-*value**	**OR (95% CI)**	***p-*value**
**AC**								
Q 1 ( ≤ 1.95)	Ref		Ref		Ref		Ref	
Q 2 (1.95-2.43)	1.756 (1.664–1.853)	<0.001	1.428 (1.350–1.511)	<0.001	1.177 (1.110–1.247)	<0.001	1.193 (1.091–1.305)	<0.001
Q 3 (2.43-3.10)	2.373 (2.253–2.498)	<0.001	1.595 (1.510–1.685)	<0.001	1.202 (1.136–1.272)	<0.001	1.205 (1.104–1.315)	<0.001
Q 4 (≥3.10)	2.962 (2.816–3.115)	<0.001	1.712 (1.621–1.807)	<0.001	1.141 (1.079–1.208)	<0.001	1.084 (0.99–1.187)	0.083
Per SD increase	1.353 (1.334–1.373)	<0.001	1.150 (1.131–1.169)	<0.001	1.013 (0.996–1.031)	0.144	0.981 (0.954–1.009)	0.178
*p* for trend	<0.001		<0.001		0.001		0.459	
**AIP**								
Q 1 ( ≤ 0.104)	Ref		Ref		Ref		Ref	
Q 2 (0.104-0.279)	1.073 (1.025–1.124)	0.003	1.038 (0.989–1.091)	0.131	0.991 (0.943–1.042)	0.725	1.012 (0.937–1.093)	0.757
Q 3 (0.279-0.466)	1.099 (1.050–1.151)	<0.001	1.037 (0.988–1.090)	0.141	0.967 (0.920–1.017)	0.194	0.990 (0.917–1.069)	0.805
Q 4 (≥0.466)	1.128 (1.077–1.180)	<0.001	1.042 (0.992–1.094)	0.10	0.942 (0.896–0.990)	0.018	0.971 (0.899–1.049)	0.453
Per SD increase	1.045 (1.029–1.062)	<0.001	1.012 (0.994–1.029)	0.184	0.974 (0.957–0.992)	0.004	0.989 (0.963–1.017)	0.437
*p* for trend	<0.001		0.127		0.011		0.367	
**CRI-I**								
Q 1 ( ≤ 2.95)	Ref		Ref		Ref		Ref	
Q 2 (2.95-3.43)	1.756 (1.664–1.853)	<0.001	1.427 (1.349–1.511)	<0.001	1.176 (1.110–1.246)	<0.001	1.191 (1.089–1.302)	<0.001
Q 3 (3.43-4.10)	2.370 (2.251–2.496)	<0.001	1.593 (1.508–1.683)	<0.001	1.201 (1.135–1.271)	<0.001	1.204 (1.103–1.314)	<0.001
Q 4 (≥4.1)	2.963 (2.18–3.116)	<0.001	1.712 (1.622–1.807)	<0.001	1.142 (1.079–1.208)	<0.001	1.082 (0.989–1.185)	0.087
Per SD increase	1.353 (1.334–1.373)	<0.001	1.150 (1.131–1.169)	<0.001	1.013 (0.996–1.031)	0.144	0.981 (0.954–1.009)	0.178
*p* for trend	<0.001		<0.001		0.001		0.466	
**CRI-II**								
Q 1 ( ≤ 1.64)	Ref		Ref		Ref		Ref	
Q 2 (1.64-1.98)	1.563 (1.483–1.647)	<0.001	1.312 (1.241–1.387)	<0.001	1.129 (1.067–1.195)	<0.001	1.121 (1.027–1.223)	0.011
Q 3 (1.98-2.44)	2.138 (2.034–2.247)	<0.001	1.522 (1.444–1.605)	<0.001	1.198 (1.134–1.265)	<0.001	1.214 (1.117–1.320)	<0.001
Q 4 (≥2.44)	2.531 (2.410–2.658)	<0.001	1.531 (1.453–1.614)	<0.001	1.089 (1.031–1.150)	0.002	1.036 (0.951–1.130)	0.419
Per SD increase	1.291 (1.272–1.310)	<0.001	1.109 (1.091–1.127)	<0.001	0.996 (0.979–1.014)	0.996	0.976 (0.949–1.003)	0.084
*p* for trend	<0.001		<0.001		0.011		0.508	
**Cholesterol index**								
Q 1 ( ≤ 35.57 mg/dl)	Ref		Ref		Ref		Ref	
Q 2 (35.57-51.8 mg/dl)	1.494 (1.417–1.574)	<0.001	1.248 (1.181–1.319)	<0.001	1.094 (1.034–1.158)	0.002	1.095 (1.003–1.194)	0.042
Q 3 (51.8-71.13 mg/dl)	2.049 (1.949–2.154)	<0.001	1.433 (1.359–1.511)	<0.001	1.169 (1.107–1.234)	<0.001	1.185 (1.090–1.289)	<0.001
Q 4 (≥71.13 mg/dl)	2.529 (2.409–2.655)	<0.001	1.519 (1.442–1.600)	<0.001	1.155 (1.094–1.218)	<0.001	1.118 (1.028–1.217)	0.009
Per SD increase	1.299 (1.280–1.318)	<0.001	1.128 (1.110–1.147)	<0.001	1.047 (1.029–1.066)	<0.001	1.037 (1.009–1.065)	0.009
*p* for trend	<0.001		<0.001		<0.001		0.008	
**LCI**								
Q 1 ( ≤ 22,238)	Ref		Ref		Ref		Ref	
Q 2 (22,238-36,041)	1.311 (1.248–1.377)	<0.001	1.139 (1.081–1.200)	<0.001	1.046 (0.991–1.103)	0.102	1.039 (0.958–1.128)	0.351
Q 3 (36,041-59,550)	1.572 (1.499–1.649)	<0.001	1.235 (1.174–1.300)	<0.001	1.092 (1.036–1.150)	0.001	1.068 (0.986–1.157)	0.105
Q 4 (≥59,550)	1.809 (1.726–1.896)	<0.001	1.278 (1.216–1.344)	<0.001	1.073 (1.019–1.129)	0.007	1.076 (0.994–1.164)	0.069
Per SD increase	1.151 (1.135–1.167)	<0.001	1.059 (1.044–1.075)	<0.001	1.019 (1.003–1.035)	0.02	1.015 (0.990–1.041)	0.247
*p* for trend	<0.001		<0.001		0.004		0.058	
**TG/HDL-C**								
Q 1 ( ≤ 1.26)	Ref		Ref		Ref		Ref	
Q 2 (1.26-1.90)	1.067 (1.019–1.117)	0.006	1.033 (0.984–1.085)	0.194	0.984 (0.936–1.034)	0.525	1.000 (0.926–1.080)	0.999
Q 3 (1.90-2.92)	1.094 (1.044–1.145)	<0.001	1.032 (0.983–1.084)	0.202	0.961 (0.914–1.011)	0.122	0.982 (0.909–1.060)	0.643
Q 4 (≥2.92)	1.125 (1.075–1.178)	<0.001	1.040 (0.990–1.092)	0.117	0.939 (0.893–0.987)	0.013	0.963 (0.891–1.040)	0.332
Per SD increase	1.039 (1.023–1.055)	<0.001	1.013 (0.997–1.030)	0.122	0.988 (0.971–1.005)	0.161	0.996 (0.970–1.023)	0.762
*p* for trend	<0.001		0.144		0.008		0.281	
**Non-HDL-C**								
Q 1 ( ≤ 107.9 mg/dl)	Ref		Ref		Ref		Ref	
Q 2 (107.9-128 mg/dl)	1.558 (1.476–1.644)	<0.001	1.267 (1.197–1.341)	<0.001	1.137 (1.072–1.204)	<0.001	1.042 (0.955–1.137)	0.354
Q 3 (128-152.3 mg/dl)	2.186 (2.077–2.301)	<0.001	1.485 (1.407–1.568)	<0.001	1.220 (1.154–1.289)	<0.001	1.144 (1.052–1.244)	0.002
Q 4 (≥152.3 mg/dl)	3.059 (2.911–3.214)	<0.001	1.708 (1.620–1.800)	<0.001	1.290 (1.222–1.363)	<0.001	1.148 (1.056–1.248)	0.001
Per SD increase	1.465 (1.443–1.488)	<0.001	1.206 (1.186–1.227)	<0.001	1.098 (1.079–1.118)	<0.001	1.061 (1.033–1.091)	<0.001
*p* for trend	<0.001		<0.001		<0.001		<0.001	

*OR, odds ratio; CI, confidence interval; Q, quartile; Model 1 adjust age and gender; Model 2 adjust Model 1 plus BMI (kg/m^2^); Model 3 adjust Model 2 plus FPG (mmol/L), ALT(U/L), AST(U/L), BUN, Scr(μmol/L) smoking status (current smoker or not), drinking status (current drinker or not), family history of diabetes (Yes or No); AC, atherogenic coefficient; AIP, atherogenic index of plasma; LCI, lipoprotein combine index; CRI, Castelli risk indices; TG, triglycerides; HDL-C, high-density lipoprotein cholesterol*.

### Predictive Value of the Cholesterol Index and Non-HDL-C for the Prevalence of Hypertension

In the multivariate logistic regression model, only the cholesterol index and non-HDL-C were identified to be independently associated with the prevalence of hypertension. Therefore, to further assess the predictive value of the cholesterol index and non-HDL-C for hypertension prevalence, the receiver operating characteristic (ROC) curve analyses were performed in overall and subgroup populations.

As presented in Table 3, in the overall population, the area under the ROC curve (AUC) for the cholesterol index and non-HDL-C on the prevalence of hypertension was 0.598 (95% CI: 0.594-0.603) and 0.620 (95% CI: 0.615-0.624), respectively. Besides, the results of subgroup analysis showed that the cholesterol index and non-HDL-C have a good predictive value for the prevalence of hypertension in women, with AUC of 0.659 and 0.684 and cut-off values of 47.94 and 134.34 mg/dl, separately. In addition, the predictive value of combining the cholesterol index and non-HDL-C for the prevalence of hypertension did not obviously improve, and the AUC remained at 0.684 (95% CI: 0.677-0.691).

**Table 3:** ROC analysis of the predictive value of cholesterol index and non-HDL-c on the prevalence of hypertension.

**Aerogenic indices**	**Group**	**Cut-off value**	**Sensitivity (%)**	**Specificity (%)**	**AUC (95%CI)**	***p*-value**
***Overall***						
Cholesterol index(mg/dl)	Overall	49.87	66	50	0.598 (0.594–0.603)	<0.001
Non-HDL-c(mg/dl)	Overall	134.73	57	60	0.620 (0.615–0.624)	<0.001
***Subgroup***						
Cholesterol index(mg/dl)	Male	49.87	65	42	0.543 (0.537–0.548)	<0.001
	Female	47.94	70	55	0.659 (0.651–0.666)	<0.001
Non-HDL-c(mg/dl)	Male	136.66	53	57	0.567 (0.561–0.573)	<0.001
	Female	134.34	61	66	0.684 (0.677–0.691)	<0.001
Cholesterol index(mg/dl)	<60 years	49.87	63	52	0.595 (0.590–0.600)	<0.001
	≥60 years	50.64	70	66	0.513 (0.504–0.522)	0.005
Non-HDL-c (mg/dl)	<60 years	127	63	54	0.613 (0.607–0.618)	<0.001
	≥60 years	137.44	61	43	0.523 (0.514–0.532)	<0.001
Cholesterol index(mg/dl)	<23 kg/m^2^	47.17	60	57	0.607 (0.598–0.615)	<0.001
	≥23 kg/m^2^	56.44	59	52	0.542 (0.536–0.547)	<0.001
Non-HDL-c(mg/dl)	<23 kg/m^2^	131.25	53	67	0.628 (0.619–0.637)	<0.001
	≥23 kg/m^2^	138.21	56	54	0.565 (0.560–0.571)	<0.001

*HDL-C, high-density lipoprotein cholesterol; ROC, receiver operating characteristic; ROC, AUC, area under the ROC curve*.

## Discussion

This study is a large-scale cross-sectional study, and the findings can be summarized as follows: 1). In Chinese adults without type 2 diabetes mellitus, the atherosclerotic indices were higher in the hypertensive group than those in the normotensive group, irrespective of the levels of hypertension. 2). SBP and DBP were positively correlated with the atherosclerotic indices. 3). The cholesterol index and non-HDL-C were independent risk factors for the prevalence of hypertension, and had a good predictive value in women.

Hypertension is not only the most important modifiable risk factor for CVD (19), but also a predictor of the extent of coronary artery disease (CAD) (20). The study by Nakanishi R et al. further clarified that hypertension is directly related to the presence and severity of coronary atherosclerosis, and adverse cardiac events (21). The principal pathophysiological mechanism is thought to be a mechanical one related to pulse pressure (22). Increased pulse pressure causes endothelial dysfunction and promotes the entry of low-density lipid cholesterol into the vessel wall, initiating the atherosclerotic process and further increasing the incidence of cardiovascular events (23, 24). As a result, the discovery of validated indicators for early hypertension identification generates a positive effect on subsequent CVD prevention and treatment. The development and progression of hypertension involves multiple pathophysiological mechanisms, including genetics, endothelial dysfunction, the renin-angiotensin-aldosterone system (RAAS), activation of the sympathetic nervous system, impaired capillary blood flow, and inflammatory mediators (25). Several previous studies have indicated that dyslipidemia is strongly associated with the prevalence of hypertension, and it is hypothesized that they are involved in some common pathophysiological mechanisms (5, 26–28). Firstly, dyslipidemia might impair endothelial function, disrupt the balance between endothelium-derived relaxing factors and endothelium-derived contracting factors, and reduce nitric oxide production, leading to endothelial cell dysfunction and disturbances in blood pressure regulation (25, 29). In addition, endothelial damage would result in loss of vasomotor activity and dysregulation of vasoconstriction in patients with dyslipidemia, thus causing a further increase in blood pressure, and creating a self-replicating vicious circle (30). Secondly, in the RAAS, angiotensin II promotes hypertension and atherosclerosis by stimulating angiotensin type 1 receptors, which increases lipid uptake in cells, free radical production, and vasoconstriction (31). Thirdly, poor lifestyle and high-fat diets could lead to obesity and dyslipidemia. In obese individuals, overproduction of adipocytokines in adipose tissue, such as leptin, induces insulin resistance, which subsequently activates the sympathetic nervous system and RAAS, and raises blood pressure (32).

The present study revealed that higher in TC and LDL-C levels, and lower in HDL-C levels in the hypertensive population, which is consistent with the results of previous study (5). Intriguingly, in our previous study, higher HDL-C levels were observed in overweight or obese hypertensive patients (28). In addition, Otsuka T et al. pointed out a U-shaped correlation between HDL-C and the risk of hypertension based on a cohort of 14,215 middle-aged men (5). Meanwhile, Yu S et al. suggested that lower or higher HDL-C levels were associated with the risk of cardiovascular events based on 10,266 Chinese adults (33). These results demonstrated that the relationship between HDL-C levels and the prevalence of hypertension in the real world may not be an absolute negative linear correlation, and it is speculated that the presence of these findings may be related to dysfunctional HDL-C. Here, it should be noted that HDL-C plays important roles in reverse cholesterol transport (RCT) and inflammation regulation, and has long been considered a protective factor against the development of cardiovascular disease (34). However, a growing body of evidence manifests that simple quantification of HDL-C levels can't adequately evaluate the function of HDL-C (35–37). In fact, HDL possesses dual properties: anti-inflammatory and pro-inflammatory properties. Moreover, under certain pathological conditions, such as disruption of the RCT capacity of Apo A-I due to oxidative mechanisms, as well as some chronic disease states, such as diabetes, HDL-C could convert to a dysfunctional state and exert pro-inflammatory effects (34). Additionally, a good example is that increasing circulating HDL cholesterol levels does not reduce cardiovascular events, while cardiovascular events occur in subjects with normal HDL-C levels (38, 39). Hence, the variability in the results of these studies might be related to the presence of dysfunctional HDL-C. However, in current clinical practice, there are no tests that are widely available to measure the composition, function, and inflammatory properties of HDL. Therefore, the measurement of HDL-C function remains a focus of future research.

Combined with the findings of this paper, the results of multivariate logistic regression suggest that among the reported atherosclerotic indices, only the cholesterol index and non-HDL-C were observed to be independently associated with the prevalence of hypertension. Among the other atherosclerosis indices, one common feature of their formulae is the use of HDL-C levels as the denominator. Initially, the establishment of these formulas was based on the protective nature of HDL-C. Considering that HDL-C function is independent of HDL-C levels, their predictive value may be weakened. In addition to that, the cholesterol index and non-HDL-C seem to be more reasonable by excluding the effect of HDL-C levels. In this case, the independent association of the cholesterol index and non-HDL-C with the prevalence of hypertension might be partly explained. Besides, our previous studies based on Bayesian models showed that TC, LDL-C, and non-HDL-C were closely related to the development of hypertension, and TC was significantly higher in the female hypertensive population than that in other subgroups (40). Furthermore, LDL-C and HDL-C are the major components that make up TC. Therefore, the association of the cholesterol index and non-HDL-C with the prevalence of hypertension may be further strengthened in women, which may explain the good prediction function of the cholesterol index and non-HDL-C for the prevalence of hypertension in women to some extent.

Gender-specific associations were demonstrated between hypertension and genetic polymorphisms, including components of the renin-angiotensin system, NO synthase, and aldosterone synthase (41). Mutations in follicle-stimulating hormone receptors were significantly related to essential hypertension in women (42). Meanwhile, the CYP 19 A1 gene, responsible for the encoding of aromatase enzyme, was associated with hypertension only in women, which was dependent on the BMI (43). Apart from that, hyperactivity of the sympathetic nervous system, coexisting with obesity, indicates this common pathophysiological mechanism in the development of hypertension (44). In addition to the increased BMI, obesity is also connected with increased plasma volume, increased exchangeable sodium, increased plasma insulin resistance and secondary hyperinsulinemia, and increased hepatic synthesis of angiotensinogen. Studies have also shown that the effect of the BMI on blood pressure is greater in women than in men (45–47). Actually, obesity and dyslipidemia usually coexist, and these mechanisms explain why dyslipidemia is more strongly associated with the prevalence of hypertension in women from the other side. Besides, there were gender differences in the frequency of immune inflammatory factors and pro/anti-inflammatory gene variants (48). Women are at increased risk for inflammatory and autoimmune diseases (49). From early post-menopause, hypertension becomes part of the metabolic syndrome (MetS), and women with MetS suffer from chronic subclinical inflammation and systemic endothelial dysfunction (49, 50). Moreover, estrogen is conducive to peripheral vascular tone, redox status, and the lipoprotein profile, and may be the most important difference between “gender dimorphism” in glucose and lipid metabolism (51, 52). Therefore, fluctuations in estrogen levels in women may affect lipids and blood pressure levels accordingly, thus further reinforcing the link between lipids and blood pressure in women.

This paper has the following strengths. Firstly, the present study is a multicenter, large sample study investigating the relationship between atherosclerotic indices and prevalence of hypertension that was rarely reported or studied with relatively small sample size in previous studies. Secondly, in our study, eight atherosclerosis indices were analyzed, and two independently associated atherosclerosis indices were obtained by regression models after adjusting for maximum covariates. At last, a predictive analysis of the independently correlated atherosclerosis indices was performed to get robust results.

Inevitably, there are also the following limitations in this paper. Firstly, the diagnosis of hypertension in this study was made through baseline blood pressure levels, which may underestimate the prevalence of hypertension. Secondly, this study was conducted based on the data provided by Chen et al. The original study design was retrospective and the study population was non-type 2 diabetic adults, while these participants were followed up at least two times during the initial 2 years and patients who developed diabetes within 2 years were further excluded from the analysis. Therefore, our study population was consistent with the original study and was a non-type 2 diabetic population. Whether the findings of this study are applicable in the diabetic population remains to be further clarified. Thirdly, other important variables such as other chronic diseases, medication history, fat distribution, and weight change (waist circumference and waist-hip ratio) were unable to be obtained from the electronic database. However, in the original study, the authors reported the participants as “apparently healthy adults”. Considering that, here, it was presumed that the participants were a relatively healthy general population. In the future, designing our study or collaborating with other researchers to improve this deficiency may be considered. Fourthly, of the 117,056 individuals, 322 participants had a TG value of 0 and 102 participants featured the HDL value of 0. Neglecting human error, it was considered that such patients were characterized with very low TG or HDL-c levels which were beyond the range of the instrument. In addition, participants with 0 values of TG and HDL-c accounted for 0.28% and 0.09% of the total number of participants, which had a negligible effect on the results. Therefore, such data were not excluded from the analysis. Fifthly, this was an observational study that provided an inference of the association between atherosclerotic indices and the prevalence of hypertension rather than causality. Therefore, further prospective studies are required to confirm that point in the future. Finally, this study was conducted in Chinese adults from 11 economically affluent regions. As the local economic status could have an impact on the implementation of primary prevention, the results might not be applied to such populations in poorer regions. In addition, the achievements of this study might not be extended to other ethnic populations due to genetic aspects.

## Conclusion

In Chinese adults without type 2 diabetes mellitus, atherosclerotic indices were significantly higher in hypertensive populations compared with those in normotensive populations, regardless of hypertension levels. Meanwhile, SBP and DBP were linearly and positively related to atherosclerotic indices. Besides, the cholesterol index and non-HDL-C were independent risk factors for the prevalence of hypertension, and they could be adopted for effectively predicting the prevalence of hypertension in women.

## Data Availability Statement

The datasets presented in this study can be found in online repositories. The names of the repository/repositories and accession number(s) can be found in the article/supplementary material.

## Ethics Statement

The studies involving human participants were reviewed and approved by Rich Healthcare Group Review Board. Written informed consent for participation was not required for this study in accordance with the national legislation and the institutional requirements.

## Author Contributions

WKC designed this topic and drafted, analyzed, and interpreted this study. JQZ and SWC provided some clinical advices. WKC, JQZ, and SWC critically reviewed the study. All authors finally agreed and approved the submitted manuscript.

## Funding

WKC was funded by China Scholarship Council (CSC No. 202009370095).

## Conflict of Interest

The authors declare that the research was conducted in the absence of any commercial or financial relationships that could be construed as a potential conflict of interest.

## Publisher's Note

All claims expressed in this article are solely those of the authors and do not necessarily represent those of their affiliated organizations, or those of the publisher, the editors and the reviewers. Any product that may be evaluated in this article, or claim that may be made by its manufacturer, is not guaranteed or endorsed by the publisher.
